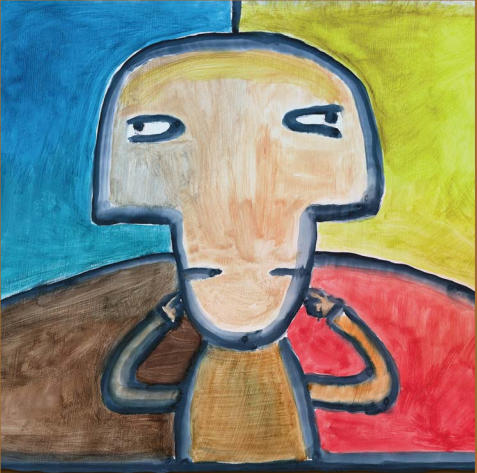# Of Two Minds: Groups Square Off on Carbon Mitigation

**DOI:** 10.1289/ehp.115-a546

**Published:** 2007-11

**Authors:** Valerie J. Brown

The highly urbanized area around Los Angeles is dotted with oil fields and refineries. Oil wells perch in yards, parking lots, even schools. The Wilmington oil field, which stretches beneath much of the land between Los Angeles and its port, as well as for miles off the coast, supplies numerous local refineries that in recent years have shut down repeatedly during power outages. Restarting the facilities often causes clouds of odorous and potentially hazardous gas to be released. After a 3 October 2007 shutdown, for example, a ConocoPhillips refinery released a cloud of “yellow, metallic dust” containing what company representatives called “a mixture of iron, copper, nickel, aluminum, carbon, and other elements,” according to the local DailyBreeze.com news service.

So when local environmental justice groups learned of plans to build a 500-megawatt power plant in nearby Carson that would sequester most of its carbon dioxide (CO_2_) emissions, it became a rallying point in their opposition to AB705, a state bill designed to set standards for carbon capture and storage (CCS). The Carson plant plans to employ integrated gasification combined cycle technology: petroleum coke (or “petcoke,” a petroleum refining by-product) would be processed to separate hydrogen and CO_2_. The former would be used to generate electricity, and 90% of the latter would be piped underground, where it would push oil reserves to the surface en route to permanent storage, in a process known as “enhanced recovery.”

The opposition to the CCS bill outlines in sharp relief what can happen when the global and the local conflict. Mitigation of global warming is a top priority for many policy analysts and technology experts who see CCS as a viable means of helping achieve that goal. But for the low-income and minority communities around Carson—a population already affected by many years of hydrocarbon extraction, processing, and transportation—the prospect of receiving yet another waste product has raised hackles.

Whether these groups can work out their differences is not clear. On a larger scale, however, the events surrounding AB705 raise key questions about the state of knowledge around CCS: Are its safety and efficacy really still in doubt? What is the state of public acceptance, understanding, and education around CCS? Are there broader environmental justice dimensions to CCS? And can the goals and values of various branches of the environmental movement be harmonized?

## The Huffman Bill

In 2006 the California State Legislature passed AB32, the Global Warming Solutions Act. The act requires the California Air Resources Board (CARB) to monitor and reduce greenhouse gas emissions by, among other strategies, adopting the “maximum technologically feasible and cost-effective reductions” in such emissions. The act also established a Global Warming Environmental Justice Advisory Committee to suggest ways for CARB to reduce greenhouse gases “while maximizing the overall societal benefits, including reductions in other air pollutants, diversification of energy sources, and other benefits to the economy, environment, and public health,” according to the committee’s mission statement.

In February 2007, Jared Huffman (D–Marin County) of the California State Assembly, California’s lower house, introduced AB705. This bill directs the California Department of Oil, Gas, and Geothermal Resources (DOGGR), in consultation with other agencies such as the California EPA, to establish regulations governing the geologic sequestration of greenhouse gases. AB705 regulations would cover underground site characterization and approval, well permitting, monitoring, modeling, remediation, drilling and construction specifications, decommissioning procedures, and property rights issues.

After introduction, the bill was referred first to the Assembly’s Utilities and Commerce Committee, then to the Natural Resources Committee. The bill—which was endorsed by the mayor of Los Angeles and the Union of Concerned Scientists, among others—was approved unanimously by the first committee, so its supporters were not prepared for opposition from environmental justice groups.

On 20 April 2007, 10 such groups wrote a letter to the Natural Resources Committee chair objecting to AB705, saying that “CO_2_ releases are deadly for communities.” As proof, they pointed to the example of Cameroon’s Lake Nyos, where a 1986 CO_2_ leak killed more than 1,700 people. The Lake Nyos incident involved a natural occurrence in which CO_2_ from volcanic sources bubbled up from the bottom of the lake and then flowed slowly downhill, asphyxiating the humans and animals that lived in the low-lying areas. The authors also pointed to concerns about CO_2_ migration into groundwater and questions about who would bear the cost of cleaning up CO_2_ leaks.

Piecing together schemes to inject toxic gases under the ground.. .in order to continue reliance on fossil fuels as our energy source is just not good policy.

– 20 April 2007 letter from 10 environmental justice groups to the Natural Resources Committee

The bill’s opponents have also suggested that implementing CCS standards and regulations would open up a slippery slope of approvals for sequestration projects, which they regard as specious solutions to global warming. “There is no currently operating project [in California] that uses carbon sequestration technology,” says Jesse Marquez, executive director of Coalition for a Safe Environment, a Wilmington-based nongovernmental organization that helped lead the opposition. “There isn’t one application pending that requires any type of carbon sequestration legislation [the Carson project has not yet applied for permits]. Because there isn’t one operating, we thought it was too premature to be establishing regulations for a technology that has not yet been thoroughly researched in terms of its environmental and public health [implications].”

Ultimately, Huffman voluntarily withdrew the bill and postponed hearings until 2008. But tabling AB705 will not stop the Carson power plant. According to George Peridas, a science fellow with the Natural Resources Defense Council (NRDC), the plant could be built and its CCS regulated under California’s existing underground injection control programs for pilot projects, but these, he says, were “never designed with large-scale sequestration in mind.” AB705, on the other hand, was an attempt to ensure that appropriate parameters are specified for industrial-scale CCS before such projects come online, Peridas says. The NRDC cosponsored AB705 and has not endorsed the Carson plant.

## A Matter of Trust

“The environmental justice people had a hard time separating CCS technology from their specific project [against the Carson plant],” says Peridas. “That rubbed them the wrong way. Perhaps justifiably, they tend to be mistrustful of industry.”

Indeed, says Marquez, CCS is merely “industry manipulation to try to circumvent growing public interest and knowledge and awareness of renewable energy portfolios. They can show that they can build a power plant and sequester the CO_2_ they generate easily and cheaply. Then they can wage a campaign to the public to say it’s still okay to build more coal plants, and now hydrogen power plants.”

Jane Williams, executive director of California Communities Against Toxics, an environmental justice group based in Rosamond that helped galvanize activists’ opposition, views AB705’s naming DOGGR as the lead agency as “an end run” around the state’s existing environmental justice committees. “DOGGR is an agency whose job it is to regulate oil extraction, not to protect public health and the environment,” Williams says. However, she adds, the CARB Global Warming Environmental Justice Advisory Committee will have jurisdiction over DOGGR’s climate change actions, so even if an end run were planned, it would not succeed. Williams is a co-chair of the CARB committee, and Marquez also is a member.

But it was not just industry that environmental justice advocates distrusted. “There was a difference between the mainstream environmental groups that are trying to find national and global solutions but fail to work with the environmental justice groups to see if we would receive any negative impacts,” Marquez says. Given this mistrust and their view of DOGGR as an inappropriate steward of environmental health, Williams says the California environmental justice groups viewed AB705 as “very averse to our interests.”

## The Science of CCS

How valid are the scientific concerns raised by the environmental justice advocates? Peridas says the claim that CCS has not been adequately studied misrepresents the state of the technology. “There are major international commercial CCS projects that have been operating for years and are showing that no CO_2_ is escaping and that there have been no detrimental effects to human health or the environment,” he says [for more information on these projects, see “Carbon Capture and Storage: Blue-Sky Technology or Just Blowing Smoke?” p. A538 this issue].

Tiffany Rau, policy and communications manager for Carson Hydrogen Power Project LLC, a partner with BP in the Carson project, adds, “This claim reflects a simple lack of knowledge of a technology that has been around for over thirty years and is used in dozens of locations around the globe. There are literally hundreds of studies about the use of CO_2_ in enhanced oil recovery, without any significant environmental or public health consequences. . . . The body of evidence is available to demonstrate that the potential that CCS will harm either the environment or human health is so distantly remote as to not pose a significant risk.”

As for the comparison with Lake Nyos, Rau explains, “CCS injects CO_2_ into the microscopic pores between the grains and crystals of subterranean rock formations, such as limestone and sandstone. The CO_2_ is not injected into large underground caverns where it can escape in large quantities and erupt to the surface in a sudden rush. . . . Catastrophic leaks are not possible [in a CCS scenario]. Slow leaks are possible, however unlikely, which is why extensive CO_2_ monitoring would accompany any CCS project.”

Marquez worries that any CO_2_ sequestered in the Wilmington oil field would immediately begin to escape through old wells. He estimates that in Wilmington alone there are 500 orphaned oil wells whose owners are unknown or cannot be located, with some 2,000–3,000 such wells in the greater area. Peridas agrees that the possibility of CO_2_ leakage from orphaned wells is a serious matter—and just the sort of thing that AB705 would require CCS projects to address during the site selection process.

There are major international commercial CCS projects that have been operating for years and are showing that no CO_2_ is escaping and that there have been no detrimental effects to human health or environment.

– George Peridas, Natural Resources Defense Council

Critics of CCS also worry that easing short-term concerns about the continued use of coal and petcoke implies approval of polluting industrial practices. Petcoke can be just as dirty to burn as coal. Depending on the type of coal or coke, its combustion can release not only CO_2_ but also toxics such as sulfur dioxide, nitrogen oxides, volatile organic compounds, and heavy metals including vanadium, nickel, and mercury. Marquez points out that even if these bad actors are stripped from air emissions, they will still need to be stored somewhere.

Peridas offers another perspective. He says it is important to distinguish between combusting petcoke and gasifying it. The former is what usually happens to the fuel when it is exported to developing countries such as China, where it releases significant quantities of harmful pollutants to the atmosphere. Gasification, which is what a plant such as the Carson facility would do, strips the pet-coke of its contaminants prior to combustion; it is not an end-of-pipe scrubbing process. “In that respect,” he says, “any gasification project that uses petcoke takes a substance off the market that elsewhere would release a vast amount of pollutants.”

Many climate policy analysts, including Peridas, agree that conservation and renewables should play a much larger part than CCS in mitigating global warming, but that CCS is also a necessary fall-back technology because of the urgency dictated by the climate problem. “Advocating for seatbelts does not mean you are in favor of accidents,” says Peridas.

## Common Ground?

In the time before the next California legislative session, there will likely be meetings among the stakeholders to find common ground regarding AB705. Peridas says he hopes to continue working with the California environmental justice community. “What I would like to see is for the environmental justice groups to sustain their level of engagement and that we have a constructive process and dialog. . . . I’m hoping we’ll have a process of engagement rather than a disconnect now and disagreement at the next contention point.”

But Williams says the wedge between the California environmental justice community and mainstream environmentalists is “a chasm” that she doesn’t see being closed unless climate change policy efforts fully involve environmental justice values in their calculus. Yet, if California’s activists can reconcile their differences, they could provide a template for a national conversation as to how global warming solutions can incorporate the goals and values of environmentalists of all stripes.

For now, AB705 will lie dormant until and unless it is revived and recommended for passage by the Assembly Natural Resources Committee in 2008. Huffman is not optimistic. “I think we just have to see if the politics are still intractable,” he says. “I don’t see the path forward for this bill.”

## Figures and Tables

**Figure:**